# Circular, “Texas Quackery”

**Published:** 1886-02

**Authors:** Alex. W. Acheson

**Affiliations:** Denison, Texas


### CIRCULAR---TEXAS QUACKERY.

DENISON, TEXAS, January, 1886.

*Dear Sir:*—As a result of inquiries thus far made, some 41 counties have reported acts evidencing incompetence or ignorance by medical impostors. Some of these are ludicrous—as, hanging patients by the heels to cure diarrhœa. Others are serious—-as, treating consumption by sticking the flesh full of splinters from a tree struck by lightning. Others again are fatal—-as shown by death from gashes cut in the spleen, through curiosity. Yet, 100 instances as bad and worse than these have been noted.

A full report is desired, embracing as many counties as possible. If you have not contributed anything yet, or, if in possession of any additional information, either in your own or adjoining counties, it will be thankfully received.

The time until the next meeting of the State Medical Association is short, and it is desired to get all these facts into shape as speedily as possible. Don't bother with a formal report, but jot down a few Texas facts and send them up. The little, trifling mistakes to which we are all liable, due to overwork, loss of sleep, or the inexperience of new practitioners, are not wanted, so much as the glaring defects and imposture of men utterly incompetent to practice medicine.

Respectfully yours, ALEX. W. ACHESON.

[ The above is published at the request of Dr. Acheson.—ED.]

